# Lesser wing of sphenoid bone dermoid cyst resection: a pediatric transorbital neuroendoscopic approach

**DOI:** 10.3171/2025.1.FOCVID24157

**Published:** 2025-04-01

**Authors:** Michael G. Brandel, Jeeho Kim, Jean-Paul J. Abboud, Vijay A. Patel, Michael L. Levy

**Affiliations:** 1 Department of Neurosurgery, University of California, San Diego/Division of Pediatric Neurosurgery, Rady Children’s Hospital, San Diego;; 2 Department of Otolaryngology, Naval Medical Center, San Diego;; 3 Department of Ophthalmology, Rady Children’s Hospital, San Diego;; 4 Department of Otolaryngology, University of California, San Diego/Division of Pediatric Otolaryngology, Rady Children’s Hospital, San Diego; and; 5 Oculofacial Surgical Arts, San Diego, California

**Keywords:** intracranial, neuroendoscopy, skull base, orbital apex

## Abstract

A 3-year-old male presented with a history of a nasal dermoid cyst with intracranial extension as well as a concomitant isolated intracranial dermoid cyst of the middle cranial fossa. Initially, at 2 years of age, he underwent successful transnasal endoscopic resection of an anterior skull base dermoid cyst. He then presented for a staged dermoid cyst resection of the right sphenoid bone at the lesser wing because of interval growth between imaging studies. A right-sided transorbital neuroendoscopic approach (TONES) was utilized for lesion resection. A gross-total resection was achieved, with no complications. Excellent neurological and cosmetic outcome was achieved.

The video can be found here: https://stream.cadmore.media/r10.3171/2025.1.FOCVID24157

## Transcript

### 0:22 Clinical History and Exam.

This patient is a 3-year-old male with a previously resected nasal dermoid cyst with intracranial extension and a known secondary dermoid cyst within the right middle cranial fossa abutting the optic nerve and temporal lobe.

With interval growth between surveillance scans, the family consented to proceed with transorbital neuroendoscopic resection and orbital reconstruction by the neurosurgery, oculoplastic, and otolaryngology teams.

Transorbital neuroendoscopic surgery was selected for its minimally invasive access to the orbit and middle cranial fossa. It was considered a more direct approach than alternatives such as pterional or supraorbital approaches.^1–5^

The patient was positioned supine under general anesthesia and intubated in the standard fashion.

The Brainlab navigation system was used to register the preoperative MRI to facial landmarks.

The right eye and face were prepped with dilute ophthalmic Betadine and draped in a sterile fashion.

The right lateral canthus was injected with 1% lidocaine with 1:100,000 epinephrine; 4-0 silk sutures were passed through the upper and lower eyelid gray lines to provide lid retraction.

### 1:47 Demonstration of Initial Lateral Canthal Incision.

An incision was made in the right lateral canthus with a number 15 blade. Using monopolar electrocautery, a lateral canthotomy and inferior and superior cantholyses were performed.

Dissection was then carried down through the orbicularis muscle and periosteum. The entire lateral orbital rim was exposed.

### 2:46 Sagittal Saw Is Used to Make Linear Cuts in the Lateral Orbital Rim.

Then, under malleable protection of the globe, a sagittal saw was used to make two linear cuts, superior to the frontozygomatic suture line and zygomatic arch.

The temporalis muscle was released off of the bone using monopolar electrocautery. The lateral orbital rim was then removed en bloc and placed on the back table for future orbital reconstruction.

Under direct, dynamic visualization with a 4-mm, 0° endoscope, the periorbita was elevated off the posterior lateral wall, exposing the orbital mass at the apex. The lesser wing of the sphenoid bone was noted to be intimately involved with the dermoid cyst.

Bone was noted to be absent laterally along the posterolateral orbit. The cyst was gently decompressed and teased off the temporal lobe dura using a combination of Fukushima suctions, B dissector, and microsurgical scissors.

The optic nerve was noted to be present medially, completely preserved, with a healthy periorbital sling protecting the orbital contents. Pressure was taken off the eye and pupillary reaction was checked every 20 minutes to ensure no mydriasis, an adequate pupillary light response, and absence of an afferent pupillary defect.

### 4:16 Piecemeal Resection of Waxy Cyst Contents.

The cyst contained both caseous material and hair fragments. The specimen was sent for surgical pathology.

Our aim was to not remove excess orbital bone in this child to minimize risk to orbital growth, which reaches full maturity by 7–8 years of age. Therefore, we drilled incrementally throughout the dissection to take only as much bone as we needed to achieve complete resection. A Stryker 2-mm drill bit and Kerrison rongeurs were used to maximize our lateral bony window and remove any microscopic disease across diseased bone until healthy temporal lobe dural pulsations were appreciated circumferentially.

Intraoperative Brainlab navigation was used to confirm gross-total resection of the cyst. Irrigation with warm saline, Floseal, and bone wax were used for hemostasis. Valsalva maneuvers were used to confirm no CSF leak or neurovascular injury; 1 cc of Kenalog was instilled into the orbit to assist with postoperative inflammation.

### 5:14 Replacement of Lateral Orbital Bone Flap and Orbital Reconstruction.

The orbital bone flap was placed back into the lateral orbit. A curvilinear KLS resorbable plate was then cut to size and secured in place with resorbable pins. A 4-0 Prolene suture was used to further reinforce the superior aspect of the plate.

Importantly, we used a resorbable plate for orbital reconstruction instead of a traditional titanium hardware, to allow for orbital growth over time. This is an important technical nuance for pediatric transorbital neuroendoscopic surgery patients.

### 5:50 Soft Tissue and Skin Closure.

The periosteum and lateral canthus were then closed with 5-0 Vicryl suture, which recreated the acute lateral canthal angle. The orbicularis muscle was then resuspended with 5-0 Vicryl suture, and the lateral canthal skin was closed with 6-0 plain gut suture.

At the conclusion of the case, antibiotic ointment was placed on the operative eye. The patient was then returned to the PICU in stable condition.

Postoperative imaging demonstrated gross-total resection of the lesion.

The patient was discharged home on postoperative day 2.

The patient remained at his neurological baseline.

In contrast to a superior eyelid incision, the lateral canthal approach allows for direct exposure of the lateral orbital rim, which in this case facilitated access to an orbital apex lesion in a young child with a small intraorbital volume and narrow working corridor. In our experience, this approach promotes safe surgical resection and yields an excellent cosmetic outcome.

In summary, the transorbital neuroendoscopic approach is safe and effective for appropriately selected cases. A three-surgeon team enables visualization, exposure, and resection.

## Disclosures

The authors report no conflict of interest concerning the materials or methods used in this study or the findings specified in this publication.

## Author Contributions

Primary surgeon: Abboud, Levy. Assistant surgeon: Brandel, Patel. Editing and drafting the video and abstract: Brandel, Kim, Patel. Critically revising the work: all authors. Reviewed submitted version of the work: all authors. Approved the final version of the work on behalf of all authors: Brandel. Supervision: Patel, Levy.

## Supplemental Information

### Patient Informed Consent

The necessary patient informed consent was obtained in this study.

## References

[1] Moe KS Bergeron CM Ellenbogen RG Transorbital neuroendoscopic surgery Neurosurgery 2010 67 3 Suppl Operative ons16 ons28 20679952 10.1227/01.NEU.0000373431.08464.43

[2] Karampouga M Terrarosa AK Patel B Anterolateral keyhole transorbital routes to the skull base: a comparative anatomical study Neurosurg Focus 2024 56 4 E3 10.3171/2024.2.FOCUS23877 38560934

[3] Houlihan LM Staudinger Knoll AJ Kakodkar P Transorbital neuroendoscopic surgery as a mainstream neurosurgical corridor: a systematic review World Neurosurg 2021 152 167 179.e4 33940270 10.1016/j.wneu.2021.04.104

[4] Ramakrishna R Kim LJ Bly RA Moe K Ferreira M Jr Transorbital neuroendoscopic surgery for the treatment of skull base lesions J Clin Neurosci 2016 24 99 104 26563603 10.1016/j.jocn.2015.07.021 PMC5955706

[5] Jeon C Hong CK Woo KI Endoscopic transorbital surgery for Meckel’s cave and middle cranial fossa tumors: surgical technique and early results J Neurosurg 2018 131 4 1126 1135 30544350 10.3171/2018.6.JNS181099

